# Early pregnancy depressive symptoms and severe maternal morbidity

**DOI:** 10.1016/j.ajogmf.2025.101830

**Published:** 2025-11-05

**Authors:** T. Caroline Bank, Janet Catov, Jiqiang Wu, Lynn M. Yee, Michelle L. Miller, Rebecca McNeil, Lara S. Lemon, Uma M. Reddy, Robert M. Silver, Kelly Zafman, George Saade, Judith Chung, Courtney D. Lynch, William A. Grobman, Kartik K. Venkatesh

**Affiliations:** Department of Obstetrics and Gynecology, The Ohio State University Columbus, OH; Department of Obstetrics and Gynecology, University of Pittsburgh, PA; Department of Obstetrics and Gynecology, The Ohio State University Columbus, OH; Department of Obstetrics and Gynecology, Northwestern University Chicago, IL; Department of Psychiatry, Indiana University Indianapolis, IN; RTI International Durham, NC; Department of Obstetrics and Gynecology, University of Pittsburgh, PA; Department of Obstetrics and Gynecology, Columbia University New York, NY; Department of Obstetrics and Gynecology, University of Utah Salt Lake City, UT; Department of Obstetrics and Gynecology, University of Pennsylvania Philadelphia, PA; Department of Obstetrics and Gynecology, Eastern Virginia Medical College Norfolk, VA; Department of Obstetrics and Gynecology, University of California, Irvine Orange, CA; Department of Obstetrics and Gynecology, The Ohio State University Columbus, OH; Department of Obstetrics and Gynecology, Brown University Providence, RI; Department of Obstetrics and Gynecology, The Ohio State University Columbus, OH

**Keywords:** maternal mental health, antenatal depression, severe maternal morbidity, delivery complications

## Abstract

**BACKGROUND:**

Maternal mental health conditions are common in pregnancy; suboptimal maternal mental health is associated with numerous adverse pregnancy outcomes, including preterm birth, hypertensive disorders of pregnancy, and maternal mortality.

**OBJECTIVE:**

The relationship between maternal mental health during early pregnancy and subsequent severe maternal morbidity (SMM) remains to be investigated. We examined whether depressive symptoms in early pregnancy were associated with SMM at delivery hospitalization.

**STUDY DESIGN:**

This was a secondary analysis of data from the Nulliparous Pregnancy Outcomes Study: Monitoring Mothers-To-Be study. In this prospective cohort, nulliparous individuals were followed from the first trimester through delivery at eight centers in the United States. The Edinburgh Postnatal Depression Scales (EPDS) was administered at 6–13 weeks’ gestation and assessed categorically at thresholds (≥10 and ≥13) that are commonly used in clinical practice. The primary outcome was SMM at delivery hospitalization, and secondarily, SMM without transfusion. Relative risk regression using a modified Poisson model with robust error variance was used and adjusted for baseline age, insurance status, tobacco use, and residential Area Deprivation Index. In secondary analyses, we further adjusted for preexisting psychiatric diagnosis and psychotropic medication exposure in early pregnancy.

**RESULTS:**

Among 8,784 nulliparas enrolled in early pregnancy (median gestational age: 12.0 weeks; interquartile range [IQR] 11.0, 13.0), 17.2% and 7.1% of individuals had an EPDS score ≥10 and ≥13, respectively. 2.3% experienced SMM and 0.5% experienced non-transfusion SMM. Having an EPDS ≥10 was associated with a greater frequency of SMM in comparison to having an EPDS <10 (3.0% vs 2.1%; relative risk [RR] 1.42; 95% confidence interval [CI] 1.02, 1.96). However, the relative risk was not significant after adjustment (adjusted relative risk [aRR] 1.17; 95% CI: 0.77, 1.77). Individuals who met the higher EPDS threshold of ≥13 had an increased risk of SMM without transfusion in unadjusted (1.1% vs 0.4%, RR 2.53, 95% CI: 1.13, 5.67) and adjusted analyses (1.1% vs 0.4%, aRR: 3.12; 95% CI: 1.11, 8.81). The above associations were similar after further adjustment for a psychiatric diagnosis and psychotropic medication exposure in early pregnancy.

**CONCLUSION:**

In a prospective US cohort of nulliparous individuals, severe early pregnancy depressive symptoms were associated with an increased risk of SMM without transfusion. Further data about whether intervention for depression in early pregnancy can affect SMM are needed.

## Introduction

Depressive symptoms are common in early pregnancy, affecting as many as one in seven pregnant individuals in the United States (U.S.).^1–3^ Mental health conditions contribute to more than 20% of pregnancy-related deaths, more than three-quarters of which are among individuals with a known history of depression and almost all of which are preventable.^4,5,6^ Furthermore, suboptimal antepartum mental health has been associated with several adverse pregnancy outcomes, including hypertensive disorders of pregnancy, preterm birth, small-for-gestational-age birth, and fetal death.^7–11^ These findings have led to public health initiatives designed to screen for, diagnose, and treat maternal mental health conditions during pregnancy.^6,12,13^

Severe maternal morbidity (SMM) includes a range of significant life-threatening complications during pregnancy, such as organ dysfunction, maternal intensive care unit admission, and need for hysterectomy.^14^ The rate of SMM in the U.S. is rising, reaching a prevalence of 179.8 per 10,000 delivery hospitalizations in 2021.^15,16^ Recent data suggest that the rate of SMM is higher among individuals with perinatal mood and anxiety disorders at delivery.^17^ However, the relationship between maternal mental health early in pregnancy and consequent SMM remains to be investigated using rigorously-collected prospective data. Understanding the influence of maternal mental health in the periconception period on SMM can inform interventions that prioritize maternal mental health to address the maternal morbidity and mortality crisis in the U.S.

The objective of the current analysis was to examine the association between early pregnancy depressive symptoms, as measured by the Edinburgh Postnatal Depression Scales (EPDS) in the first trimester and SMM at delivery hospitalization. We hypothesized that screening positive for more symptoms of antenatal depression would be associated with an increased risk of SMM.

## Materials and methods

### Setting

This is a secondary analysis of the Nulliparous Pregnancy Outcomes Study: Monitoring Mothers-to-Be (nuMoM2b), a prospective cohort study designed to assess the influence of maternal and environmental factors on pregnancy outcomes.^18^ Nulliparous pregnant individuals were enrolled at eight U.S. medical centers from October 2010 to September 2013, and data were collected prospectively by standardized patient interviews, surveys, and health record abstraction at up to three time points during pregnancy and at delivery.^18^ The institutional review board at each participating site approved the study, and participants provided written informed consent prior to participation.

### Participants

Key inclusion criteria for cohort enrollment included: a singleton pregnancy with an estimated gestational age from 6 0/7 to 13 6/7 weeks, no prior delivery at 20 weeks of gestation or later, and intention to deliver at a participating hospital. Exclusion criteria included: age younger than 13 years, a history of three or more pregnancy losses, donor oocyte pregnancy, planned pregnancy termination, fetal malformations likely to be lethal, known fetal aneuploidy, previous enrollment in the study, and inability to provide informed consent. For this analysis, individuals who did not complete the EPDS at the first study visit were excluded.

### Exposure

The exposure was antenatal depressive symptoms measured by the EPDS at study visit 1 between 6 weeks 0 days and 13 weeks 6 days. Measurement of depressive symptoms at that time is consistent with current recommendations to screen for risk of antenatal depression in early pregnancy.^6^ The EPDS is a standardized and validated self-administered questionnaire that consists of 10 short statements.^6,19^ We measured the EPDS at two thresholds commonly used in clinical practice: an EPDS score ≥ 10 has a high sensitivity for a clinical diagnosis of antenatal depression^19^; and a higher threshold of an EPDS score ≥13 is a more specific cutoff for severe antenatal depression.^19^

### Outcome

The primary outcome was any SMM at delivery hospitalization. SMM without blood transfusion was secondarily assessed, as blood transfusion has often been excluded from prior analyses assessing SMM, and because transfusion is the most prevalent SMM indication and may not reflect severe clinical morbidity.^20^ In this cohort, trained research nurses recorded clinical diagnoses, procedures, and common complications for each delivery hospitalization. A study author (XXX), blinded to participant EPDS score, coded obstetric outcomes according to the U.S. Centers for Disease Control and Prevention (CDC) guidance for identification of SMM.^21^ The CDC has selected 21 indicators for severe life-threatening diagnoses and related procedures recorded at delivery hospitalization discharge (Supplementary Table 1).^16^ SMM was categorized by a composite binary indicator (yes or no) for whether any of the 21 indicators was present.

### Covariates

Covariates were selected *a priori* for inclusion in multivariable analysis based on a directed acyclic graph and informed by the hypothesized relationships between depression symptoms and SMM (Supplementary Figure 1).^22^ Models were adjusted for maternal age (continuous), insurance status (public or private), tobacco use (yes or no), and residential Area Deprivation Index (quartiles) as a measure of neighborhood-level socioeconomic status, selected *a priori*.^23^ In secondary analyses, we also adjusted for whether an individual had a diagnosis of a current mental health condition (depression, anxiety, bipolar disorder, and schizophrenia), as identified via chart review at visit 1, and if so, was taking psychotropic medications (inclusive of any use of one or more of the following medication classes: monoamine oxidase inhibitors, tricyclic antidepressants, selective serotonin reuptake inhibitors, serotonin-norepinephrine reuptake inhibitors, and norepinephrine-dopamine reuptake inhibitors). Mental health conditions and psychotropic medication exposure were assessed as a single trichotomous mental health covariate (no diagnosis/no medication, yes diagnosis/no medication, yes diagnosis/yes medication).

### Statistical analysis

We compared the frequency of participant characteristics by EPDS scores (≥10 versus <10, and ≥13 versus <13) using chi-square tests for categorical variables and Wilcoxon rank sum tests for continuous variables. Relative risk regression using a modified Poisson model was used to estimate the unadjusted and adjusted relative risk (RR, aRR) and 95% confidence intervals (95% CI) between an EPDS score at or above the cutoff and SMM after adjusting for covariates. Imputation for missing covariate data was performed using Multiple Imputation by Chained Equations or MICE (n=30 imputations) and estimates were combined using Rubin’s rule. All statistical analyses were performed using STATA (StataCorp, LLC, version 16.1, College Station, TX) and R statistical software version 4.2.0 (R Foundation for Statistical Computing).

## Results

Among 10,038 nulliparous participants, 1254 (12%) did not complete the EPDS in the first trimester. Thus, the final analytic sample included 8,784 (88%) individuals. Age, insurance status, racial and ethnic self-identification, educational attainment, household income, and living with diabetes and obesity varied by individuals who completed the early pregnancy EPDS versus those who were excluded (*P*<.05 for all) (Supplementary Table 2).

At enrollment, the median age of participants was 27 years (IQR 23.1, 31.0) (Table 1). Twenty-seven percent had Medicaid health insurance, and 7.6% had an educational attainment of a high school diploma or less. Twenty-one percent had a BMI ≥30 kg/m^2^, 1.5% had pregestational diabetes, 2.5% had chronic hypertension, and 17.7% reported tobacco use. Overall, 13.3% reported baseline psychiatric diagnosis and 4.6% of these individuals reported psychotropic medication use.

The mean EPDS was 5.0 (SD 3.0, 8.0), and 17.2% of individuals had an EPDS ≥10 and 7.1% had an EPDS ≥13. In comparison to those with an EPDS <10, individuals with an EPDS ≥10 were older, more likely to be Medicaid-insured, have lower educational attainment and household income, have higher or worse ADI, use tobacco, and self-identify as a minoritized race and ethnicity (*P*<.05 for all) (Table 1). Individuals with an EPDS ≥10 also were more likely to have a current mental health diagnosis at enrollment (22.4% vs 11.4%, *P*<.05) and to report psychotropic medication use with a mental health diagnosis (9.4% vs 3.6%, *P*<.05). These results were similar when using a higher EPDS screening threshold of ≥13 vs <13 as the reference.

**FIGURE 1:**
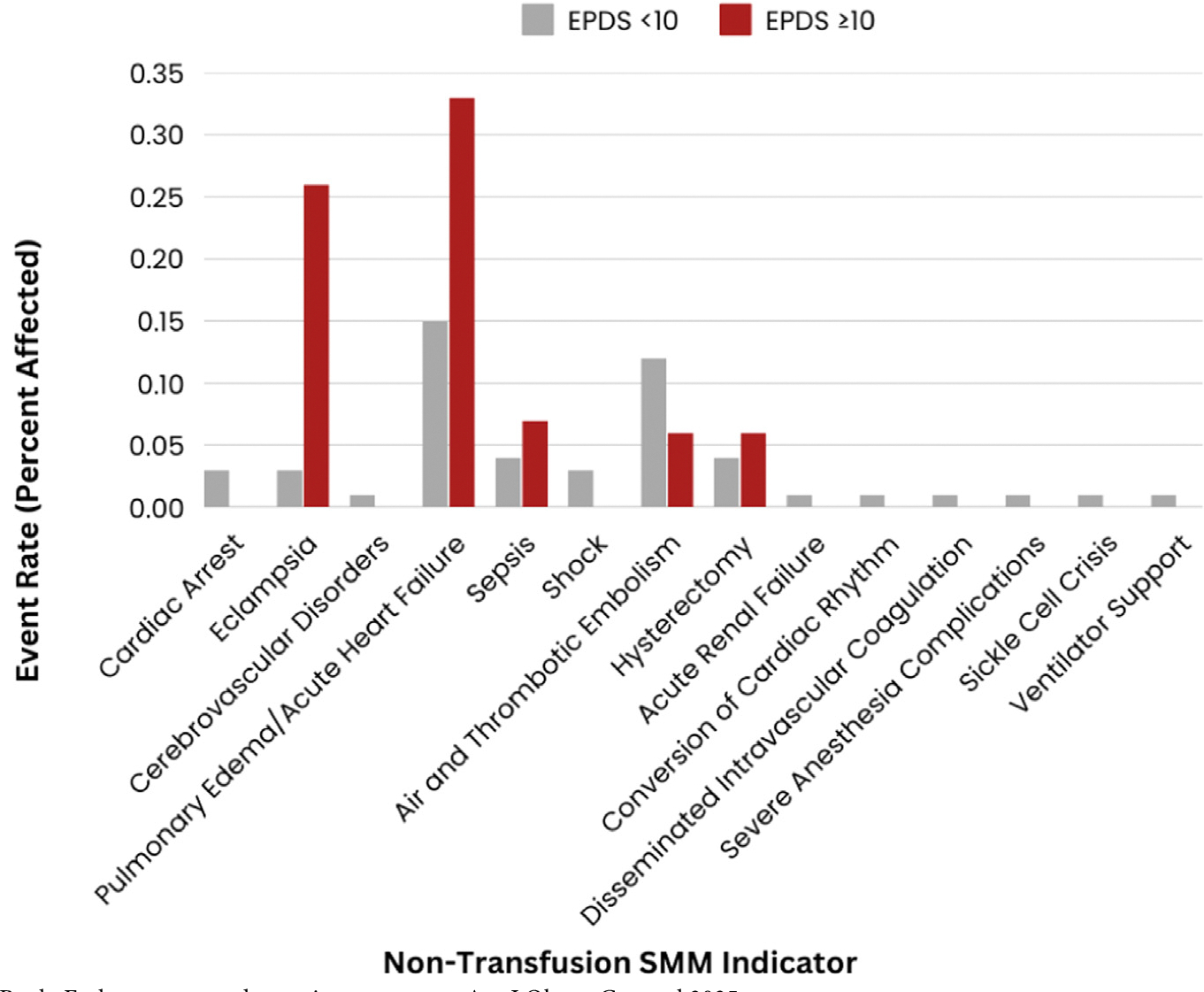
Rate of non-transfusion SMM among individuals with an EPDS <10 vs ≥ 10 Bank. Early pregnancy depressive symptoms. Am J Obstet Gynecol 2025.

**FIGURE 2:**
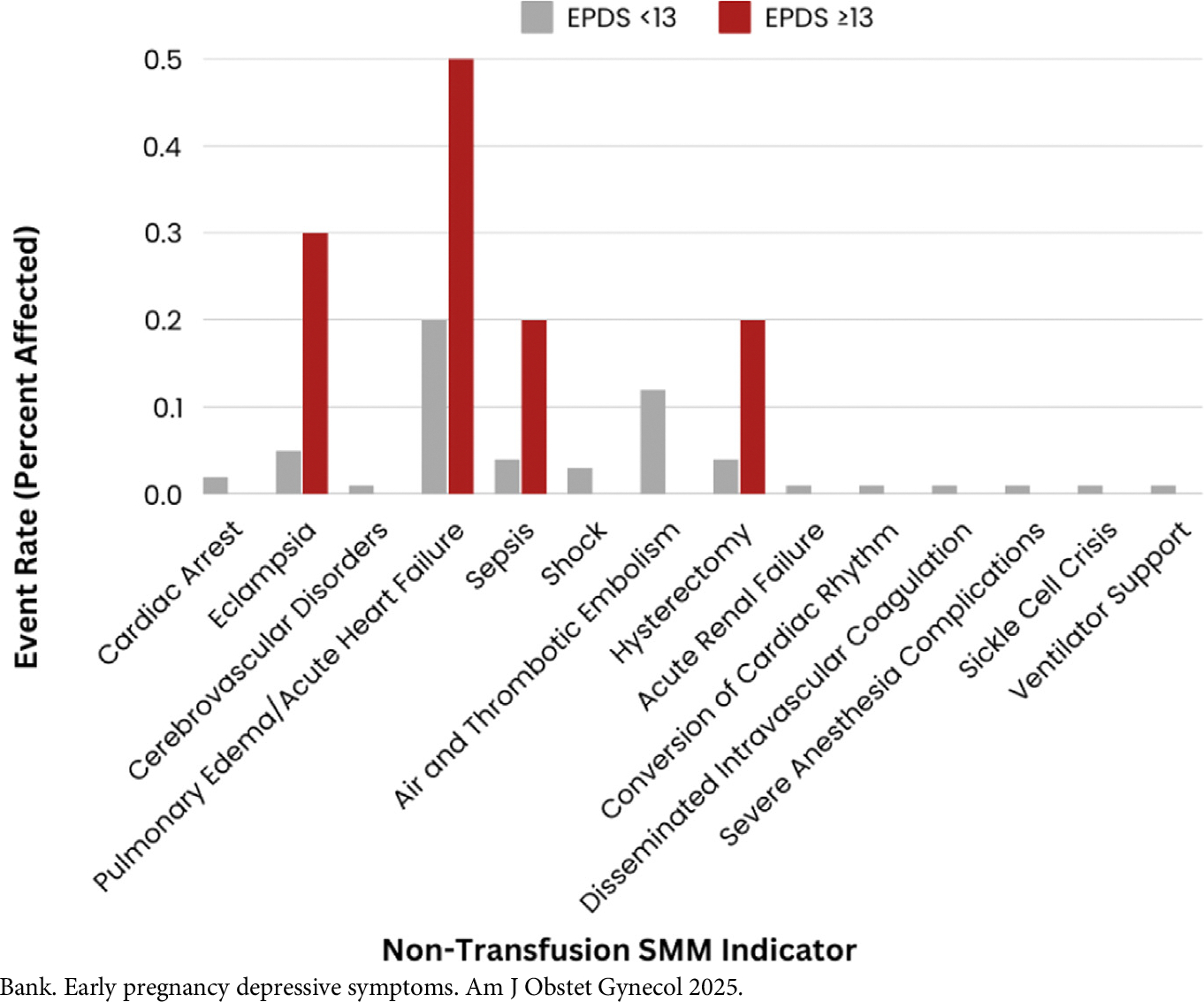
Rate of non-transfusion SMM among individuals with an EPDS <13 vs ≥ 13 Bank. Early pregnancy depressive symptoms. Am J Obstet Gynecol 2025.

Overall, 2.3% (n=198) individuals experienced SMM and 0.5% (n=43) individuals experienced SMM without transfusion. After transfusion (n=162, 81% of total SMM events), the most frequent SMM diagnoses (not mutually exclusive) were pulmonary edema/acute heart failure (n=16, 8%), air and thrombotic embolism (n=10, 5%), eclampsia (n=6, 3%), sepsis (n=4, 2%), and hysterectomy (n=4, 2%) (Figures 1 and 2).

Individuals with an early pregnancy EPDS ≥10 were at greater risk of SMM (3.0% vs 2.1%; RR: 1.42; 95% CI: 1.02, 1.96) in unadjusted analysis, although this association was no longer significant in adjusted analyses (aRR 1.17; 95% CI: 0.77, 1.77). Similarly, there was no association between early pregnancy EPDS ≥10 and SMM without transfusion. At a higher screening threshold, there was no association between EPDS ≥13 and SMM. However, individuals with an EPDS ≥13 had a significantly increased risk of SMM without transfusion both in unadjusted (1.1% vs 0.4%, RR 2.53, 95% CI: 1.13, 5.67) and in adjusted analyses (1.1% vs 0.4%, aRR: 3.12; 95% CI: 1.11, 8.81) (Table 2).

In secondary analyses, the above association between an EPDS of ≥13 and SMM without transfusion held after further adjusting for a mental health condition or psychotropic medication use (1.1% vs 0.4%; aRR: 3.37, 95% CI: 1.08, 10.53) (Table 2).

## Comment

### Principal findings

In a prospective cohort of nulliparous individuals from across the US, the presence of more severe early pregnancy depressive symptoms, as measured by an EPDS score ≥13 vs <13, was associated with an increased risk of SMM without transfusion at delivery hospitalization. However, there was no association between quantity of depressive symptoms at a lower threshold (EPDS score ≥10 vs <10) and SMM with or without transfusion.

### Results in the context of what is known

These findings, demonstrating an association between depressive symptoms in the first trimester and SMM, extend what is currently known about the association between antenatal depression and adverse neonatal (preterm birth, small-for-gestational-age birth, NICU admission, and low Apgar scores)^24–29^ and maternal (hypertensive disorders of pregnancy, placental abruption, and cesarean delivery) outcomes.^30–32^ Other maternal mental health conditions, such as bipolar disorder and schizophrenia, also have been associated with pregnancy complications such as hemorrhage, venous thromboembolism, and maternal admission to an intensive care unit.^33,34^ A cross-sectional analysis of the National Inpatient Sample relying on administrative billing codes found that perinatal mood and anxiety disorders were associated with higher rates of SMM and maternal mortality. In addition, that study found an association between mental health diagnostic codes and increased healthcare utilization at delivery hospitalization, including more hospital transfers, longer length of stay, and higher delivery-related costs.^17^

In the current study, the association between antenatal depressive symptoms and SMM without transfusion remained even after adjustment for mental health conditions and psychotropic medication exposure. Emerging data suggest that treatment of depression in early pregnancy, including behavioral intervention or medication, may decrease the risk of adverse pregnancy outcomes.^8,35^ Taken together, these findings highlight the importance of studying whether adequate and timely mental health treatment can reduce non-psychiatric maternal morbidity.

There are several mechanisms through which suboptimal maternal mental health may contribute to an increased risk of SMM. Depressive symptoms are associated with disruptions in immune, neuroendocrine, metabolic, and sympathetic nervous system activity.^36–39^ Depressive symptoms negatively affect positive healthcare-seeking behaviors, including diet quality, sleep, and physical activity.^40^ Pregnant individuals with more antenatal depressive symptoms also are less likely to engage in prenatal care, which may limit early detection and treatment of pregnancy complications.^13,41^ In addition, several social factors, including intimate partner violence and incarceration, have previously been associated with both perinatal depression and risk of SMM.^42–49^

### Clinical implications

These findings suggest the need for further prospective assessment of whether screening for depressive symptoms early in pregnancy, in addition to its other health benefits, may help to identify those at highest risk of SMM. In this analysis, entering pregnancy with fewer depressive symptoms was associated with reduced risk of adverse outcomes. The persistent association between more severe depressive symptoms and SMM without transfusion after adjustment for mental health conditions and psychotropic medication use suggests that regardless of underlying diagnosis or prescription of psychotropic medication in early pregnancy, experiencing fewer depressive symptoms correlates with improved outcomes. However, this epidemiologic association should be explored further in prospective studies that specifically focus on the timing and effects of diagnosis and treatment of maternal mental health conditions on SMM.

### Research implications

Although this analysis suggests that depressive symptoms in early pregnancy are associated with increased risk of SMM, further research is needed to understand both the underlying mechanism behind this association in order to further understand whether interventions that improve access and uptake of maternal mental health resources alter this risk.^39^ It is also critical to study risk-assessment approaches for SMM that incorporate maternal mental health. Given both adverse mental health conditions and SMM are more frequent among individuals who self-identify as being of minoritized race or ethnicity and with adverse social determinants of health, understanding and improving equitable implementation of depression screening and treatment should be considered as part of research initiatives designed to improve equitable maternal care delivery.

### Strengths and limitations

A strength of the current study is that these data were prospectively collected from multiple, geographically diverse institutions across the U.S., increasing the generalizability of these findings. In addition, this study analyzed depressive symptoms in early pregnancy – prior to the occurrence of SMM – rather than at the time of delivery hospitalization. This identifies an opportunity to investigate risk-reducing interventions that can be employed between identification of depressive symptoms in early pregnancy and development of SMM. Finally, this analysis used diagnoses and procedures prospectively abstracted using a standardized study protocol. This may more accurately capture SMM than ICD-10 codes, which are prone to misclassification.^50,51^ Specifically, blood transfusion has frequently been excluded from SMM analyses due to frequent inaccuracy of administrative data; in this cohort blood transfusion was identified based on chart review by research staff.

A limitation of this study was these data were limited to nulliparous individuals who initiated prenatal care in the first trimester and chose to participate in a prospective cohort. Access to prenatal care and the potential impact of delayed or infrequent care were not assessed. While adjusted analyses controlled for individual and neighborhood-level social determinants of health in addition to baseline psychiatric morbidity, there are additional factors that were not accounted for in this analysis that may affect the association of depressive symptoms and SMM, such as social support, incarceration, illicit drug use, and intimate partner violence. Individuals who experienced more adverse individual-level adverse social determinants, and in particular Medicaid insurance, were more likely to excluded from this analysis due to missing exposure (ADI) and outcome (SMM) data, and these excluded individuals are likely more likely to experience adverse maternal mental health and SMM. Hence, the observed association may be biased to the null (i.e., non-differential misclassification). In addition, all preexisting mental health conditions were grouped together in this analysis, despite the heterogeneity of mental health conditions and their potential treatment.

## Conclusions

In a prospective, geographically diverse, nulliparous U.S. cohort, an EPDS score ≥13 in the first trimester was associated with an increased risk of SMM without transfusion at delivery hospitalization. Understanding the influence of maternal mental health in early pregnancy on SMM can inform future interventions that prioritize addressing maternal mental health to address the maternal morbidity and mortality crisis in the U.S.

## Supplementary Material

Supplementary

Supplementary material associated with this article can be found in the online version at doi:10.1016/j.ajogmf.2025.101830.

## Figures and Tables

**TABLE 1:** Socio-demographic and clinical characteristics of nulliparous pregnant individuals overall and by EPDS scores

Variable	Overall N=8784	EPDS < 10 N=7273	EPDS ≥ 10 N=1511	EPDS < 13 N=8158	EPDS ≥ 13 N=626
EPDS score, mean (SD)	5.0 (3.0, 8.0)	4.0 (2.0, 6.0)	12.0 (11.0, 14.0)^a^	5.0 (2.0, 7.0)	15.0 (14.0, 17.0)^a^
Age, years					
Median (IQR)	27.0 (23.0, 31.0)	28.0 (23.0, 31.0)	24.0 (20.0, 29.5)	28.0 (23.0, 31.0)	23.0 (20.0, 28.8)
≤17	198 (2.3)	139 (1.9)	59 (3.9)	171 (2.1)	27 (4.3)
18–34	7,780 (88.6)	6,440 (88.5)	1,340 (88.7)	7,226 (88.6)	554 (88.5)
35–39	687 (7.8)	592 (8.1)	95 (6.3)	645 (7.9)	42 (6.7)
≥40	119 (1.4)	102 (1.4)	17 (1.1)^a^	116 (1.4)	3 (0.5)^a^
Medicaid insurance (n=8728)					
Yes	2353 (27.0)	1731 (23.9)	622 (41.6)	2048 (25.2)	305 (49.8)
No	6375 (73.0)	5503 (76.1)	872 (58.4)^a^	6068 (74.8)	307 (50.2)^a^
Self-reported race and ethnicity					
Non-Hispanic White	5475 (62.3)	4700 (64.6)	775 (51.3)	5163 (63.3)	312 (49.8)
Non-Hispanic Black	1119 (12.7)	834 (11.5)	285 (18.9)	984 (12.1)	135 (21.6)
Hispanic	1386 (15.8)	1071 (14.7)	315 (20.8)	1256 (15.4)	130 (20.8)
Non-Hispanic Asian	359 (4.1)	308 (4.2)	51 (3.4)	344 (4.2)	15 (2.4)
Not specified	445 (5.1)	360 (4.9)	85 (5.6)^a^	411 (5.0)	34 (5.4)^a^
Education (n=8783)					
High school or less	668 (7.6)	465 (6.4)	203 (13.4)	553 (6.8)	115 (18.4)
Some college	2645 (30.1)	1999 (27.5)	646 (42.8)	2361 (28.9)	284 (45.4)
College graduate	3406 (38.8)	2941 (40.4)	465 (30.8)	3236 (39.7)	170 (27.2)
Graduate degree	2064 (23.5)	1867 (25.7)	197 (13.0)^a^	2007 (24.6)	57 (9.1)^a^
Area Deprivation Index score, median (IQR) (n=8453)	38.0 (18.0, 69.0)	35.0 (17.0, 65.0)	47.0 (25.0, 81.0)^a^	37.0 (18.0, 67.0)	54.0 (27.0, 86.0)^a^
Tobacco use (N=8781)					
Yes	1555 (17.7)	1096 (15.1)	459 (30.4)	1328 (16.3)	227 (36.3)
No	7226 (82.3)	6174 (84.9)	1052 (69.6)^a^	6827 (83.7)	399 (63.7)^a^
Body mass index category, kg/m^2^ (n=8656)					
Underweight	201 (2.3)	154 (2.1)	47 (3.2)	179 (2.2)	22 (3.6)
Normal weight	4426 (51.1)	3737 (52.2)	689 (46.2)	4153 (51.6)	273 (44.4)
Overweight	2150 (24.8)	1769 (24.7)	381 (25.6)	1992 (24.8)	158 (25.7)
Obesity	1031 (11.9)	836 (11.7)	195 (13.1)	947 (11.8)	84 (13.7)
Severe obesity	848 (9.8)	669 (9.3)	179 (12.0)^a^	770 (9.6)	78 (12.7)^a^
Household income and size relative to the U.S. poverty level (n=7248)					
<130%	5118 (70.6)	4545 (74.3)	573 (50.6)	4926 (72.5)	192 (41.9)
130 to 350%	1021 (14.1)	794 (13.0)	227 (20.1)	923 (13.6)	98 (21.4)
>350%	1109 (15.3)	777 (12.7)	332 (29.3)^a^	941 (13.9)	168 (36.7)
Pregestational diabetes (n=8674)					
Yes	127 (1.5)	103 (1.4)	24 (1.6)	119 (1.5)	8 (1.3)
No	8547 (98.5)	7087 (98.6)	1460 (98.4)	7945 (98.5)	602 (98.7)
Chronic hypertension (n=8604)					
Yes	217 (2.5)	175 (2.5)	42 (2.9)	201 (2.5)	16 (2.6)
No	8387 (97.5)	6962 (97.5)	1425 (97.1)	7795 (97.5)	592 (97.4)
Mental health diagnosis and psychotropic medication use (n=8513)					
Neither diagnosis nor medication	6990 (82.1)	5993 (85.0)	997 (68.2)	6605 (83.5)	385 (63.6)
Yes diagnosis, no medication	1132 (13.3)	805 (11.4)	327 (22.4)	983 (12.4)	149 (24.6)
Yes diagnosis, yes medication	391 (4.6)	253 (3.6)	138 (9.4)^a^	320 (4.0)	71 (11.7)^a^

Chi-square test was used to compare categorical variables and Wilcoxon rank sum test for continuous variables.

a *P*<.05 for assessed characteristics.

**TABLE 2:** Association between antepartum depressive symptoms in early pregnancy and severe maternal morbidity (SMM) at delivery hospitalization in nulliparous individuals

	SMM n, % (Row Percentage)		Unadjusted and adjusted analyses		
			Unadjusted analysis (95% CI)	Adjusted analysis	Sensitivity Analysis (95% CI)^a^
	No N=8586	Yes N=198	Risk ratio (95% CI)	Adjusted risk ratio (95% CI)^a^	Adjusted risk ratio (95% CI)^a^
SMM					
EPDS ≥10					
No	7,120	153 (2.1)	1.00	1.00	1.00
Yes	1,466 (97.0)	45 (3.0)	**1.42 (1.02, 1.96)**	1.17 (0.77, 1.77)	1.19 (0.78, 1.82)
EPDS ≥13					
No	7,981 (97.8)	177 (2.2)	1.00	1.00	1.00
Yes	605 (96.6)	21 (3.4)	1.55 (0.99, 2.41)	1.42 (0.81, 2.47)	1.39 (0.79, 2.43)
SMM without transfusion	No N=8,741	Yes N=43			
EPDS ≥10					
No	7,242 (99.6)	31 (0.4)	1.00	1.00	1.00
Yes	1,499 (99.2)	12 (0.8)	1.86 (0.96, 3.62)	2.03 (0.91, 4.55)	2.21 (0.96, 5.12)
EPDS ≥13					
No	8,122 (99.6)	36 (0.4)	1.00	1.00	1.00
Yes	619 (98.9)	7 (1.1)	**2.53 (1.13, 5.67)**	**3.12 (1.11, 8.81)**	**3.37 (1.08, 10.53)**

a Model adjusted for baseline age, insurance status, tobacco use, and Area Deprivation Index.;

b Above model also adjusted for: mental health conditions and/or psychotropic medication use.; Bolded results are statistically significant (*P*<.05).; N=8784

## AJOG MFM at a Glance

### Why was this study conducted?

The relationship between maternal mental health in early pregnancy and consequent severe maternal morbidity (SMM) at delivery remains to be investigated using rigorously collected prospective data. Understanding the influence of maternal mental health on SMM can inform future interventions that prioritize attending to maternal mental health to address the maternal morbidity and mortality crisis in the U.S.

### What are the key

An Edinburgh Postnatal Depression Scale score ≥13 in early pregnancy was associated with an increased risk of severe maternal morbidity (without transfusion) at delivery. There was no association between an Edinburgh Postnatal Depression Scale score ≥10 and the risk of severe maternal morbidity.

### What does this study add to what is already known?

This study demonstrates an association between maternal mental health in early pregnancy and subsequent severe maternal morbidity. This finding suggests an opportunity to identify individuals at an increased risk of severe maternal morbidity and mortality through depression screening in early pregnancy.
