# Supplementary material for: Early pregnancy depressive symptoms and severe maternal morbidity
Source: Am J Obstet Gynecol MFM. Author manuscript; Available in PMC 2026 May 19. (PMC13185947; doi:10.1016/j.ajogmf.2025.101830)
Supplement: Supplementary [file NIHMS2168126-supplement-Supplementary.docx]

| **Supplementary Table 1. Indicators of Severe Maternal Morbidity^16^** |
| --- |
| Acute Myocardial Infarction |
| Aneurysm |
| Acute Renal Failure |
| Acute Respiratory Distress Syndrome |
| Amniotic Fluid Embolism |
| Cardiac Arrest/Ventricular Fibrillation |
| Conversion of Cardiac Rhythm |
| Disseminated Intravascular Coagulation |
| Blood Transfusion |
| Eclampsia |
| Heart Failure/Arrest During Surgery |
| Puerperal Cerebrovascular Disorders |
| Pulmonary Edema/Acute Heart Failure |
| Severe Anesthesia Complications |
| Sepsis |
| Shock |
| Sickle Cell Disease With Crisis |
| Air and Thrombotic Embolism |
| Hysterectomy |
| Temporary Tracheostomy |
| Ventilation |

| **Supplementary Table 2. Socio-demographic and clinical characteristics between overall sample and final analytic sample** | | | |
| --- | --- | --- | --- |
| **Variable** | **Overall**  **N = 10,038** | **Excluded**  **N=1,254** | **Included**  **N=8,784** |
|  | **N (%)** | **N (%)** | **N (%)** |
| Age, years, median (IQR) (N=10,028)  <17  18-34  35-39  ≥40 | 27.0 (22.0, 31.0)  244 (2.4)  8,852 (88.3)  788 (7.9)  144 (1.4) | 25.0 (21.0, 31.0)  46 (3.7)  1,072 (86.2)  101 (8.1)  25 (2.0) | 27.0 (23.0, 31.0)  198 (2.3)  7,780 (88.6)  687 (7.8)  119 (1.4)* |
| Medicaid insurance (N=9,959)  Yes  No | 2,854 (28.7)  7,105 (71.3) | 501 (40.7)  730 (59.3) | 2,353 (27.0)  6,375 (73.0)* |
| Race and ethnicity (N=10,028)  Non-Hispanic White  Non-Hispanic Black  Hispanic  Non-Hispanic Asian  Other | 5,989 (59.7)  1,418 (14.1)  1,700 (17.0)  407 (4.1)  514 (5.1) | 514 (41.3)  299 (24.0)  314 (25.2)  48 (3.9)  69 (5.5) | 5,475 (62.3)  1,119 (12.7)  1,386 (15.8)  359 (4.1)  445 (5.1)* |
| Education (N=10,020)  High school or less  Some college  College graduate  Graduate degree | 816 (8.1)  3,119 (31.1)  3,777 (37.7)  2,308 (23.0) | 148 (12.0)  474 (38.3)  371 (30.0)  244 (19.7) | 668 (7.6)  2,645 (30.1)  3,406 (38.8)  2,064 (23.5)* |
| Tobacco use (N=10,018)  Yes  No | 1,782 (17.8)  8,236 (82.2) | 227 (18.4)  1,010 (81.6) | 1,555 (17.7)  7,226 (82.3) |
| Body mass index, kg/m^2^ (N=9,812)  Underweight  Normal weight  Overweight  Obese  Severely obese | 230 (2.3)  4,966 (50.6)  2,444 (24.9)  1,170 (11.9)  1,002 (10.2) | 29 (2.5)  540 (46.7)  294 (25.4)  139 (12.0)  154 (13.3) | 201 (2.3)  4,426 (51.1)  2,150 (24.8)  1,031 (11.9)  848 (9.8)* |
| Household income and size relative to the U.S. poverty level (N=8,128)  <130%  130 to 350%  >350% | 5,662 (69.7)  1,169 (14.4)  1,297 (16.0) | 544 (61.8)  148 (16.8)  188 (21.4) | 5,118 (70.6)  1,021 (14.1)  1,109 (15.3)* |
| Pregestational diabetes (N=9,566)  Yes  No | 9,415 (98.4)  151 (1.6) | 868 (97.3)  24 (2.7) | 8,547 (98.5)  127 (1.5)* |
| Chronic hypertension (N=9,469)  Yes  No | 243 (2.6)  9,226 (97.4) | 26 (3.0)  839 (97.0) | 217 (2.5)  8,387 (97.5) |
| Chi-square test was used to compare categorical variables and Wilcoxon rank sum test for continuous variables.  p<0.05 for all assessed characteristics above. | | | |

**Supplementary Figure 1. Directed Acyclic Graph.**
